# Evaluation of Facial Beauty Using Anthropometric Proportions

**DOI:** 10.1155/2014/428250

**Published:** 2014-02-20

**Authors:** Jovana Milutinovic, Ksenija Zelic, Nenad Nedeljkovic

**Affiliations:** ^1^Jovana Milutinovic, Nenad Nedeljkovic, Clinic of Orthodontics, University of Belgrade, 11000 Belgrade, Serbia; ^2^Ksenija Zelic, Laboratory for Anthropology, Department of Anatomy, School of Medicine, University of Belgrade, 11000 Belgrade, Serbia

## Abstract

The improvement of a patient's facial appearance is one of the main goals of contemporary orthodontic treatment. The aim of this investigation was to evaluate the difference in facial proportions between attractive and anonymous females in order to establish objective facial features which are widely considered as beautiful. The study included two groups: first group consisted of 83 Caucasian female subjects between 22 and 28 years of age who were selected from the population of students at the University of Belgrade, and the second group included 24 attractive celebrity Caucasian females. The en face facial photographs were taken in natural head position (NHP). Numerous parameters were recorded on these photographs, in order to establish facial symmetry and correlation with the ideal set of proportions. This study showed significant difference between anonymous and attractive females. Attractive females showed smaller face in general and uniformity of the facial thirds and fifths, and most of the facial parameters meet the criteria of the ideal proportions.

## 1. Introduction

Specialists in charge of the facial region are noticing a growing demand for the treatment mainly based on aesthetic principles. As a result, orthodontists and maxillofacial surgeons should have a great understanding for quantitative, objective facial features, which are widely considered as attractive and beautiful [1, 2].

Recently, many linear and angular measures of soft tissue profile and variety of cephalometric analyses were developed to determine ideal proportions [1–4].

When it comes to making a positive first impression, having an aesthetically pleasing face, including an attractive smile, ranks first among all factors.

Today's society is overwhelmed with the importance of being attractive through a variety of media. That fact brings facial standards together with the perception of beauty associated with a sense of social acceptance [5–9]. It was shown in the results of many studies that confidence is closely related to physical appearance [10–12].

Today's most common reason for seeking orthodontic treatment is enhancement of facial beauty through orthodontic and orthognathic procedures. Unlike the 1980, when 25% of patients required orthodontic treatment for aesthetic reasons, today this percentage has risen to over 75%, which means that 3 out of 4 patients specifically request an improvement of facial appearance [13].

Beauty is not an exact science but according to some plastic surgeons there is a specific proportion system that includes facial height, width, and symmetry. However, the definition of an attractive and beautiful face is subjective, with many included factors—social, cultural, ethnic, and age [10].

The beauty of the person's face is determined by the harmony of proportions and symmetry [12]. Ideal proportions are directly related to the so-called divine proportions and the most important value in relation to these proportions is 1 : 1.618 [11–18].

The knowledge of divine proportion exists since ancient Greek sculptor Phidias, and it was firstly scientifically described by Filius Bonacci, discoverer of the numerical value of the divine proportions [19]. More relevant to the dental profession as well as the medical profession (such as plastic surgeons) are the divine proportions of the human face.

This particularly applies to the orthodontic treatment given that the objectives to be achieved at the end of the treatment are not only functional stability, but also facial esthetic improvement [20].

The aim of our paper was to try to establish objective facial features which are widely considered as beautiful. Therefore, specific aims of this paper were the following:

1. to compare the facial proportions of two groups of females (anonymous and attractive) in order to establish the difference between them,
2. to determine the deviation from the values of ideal proportions (ratio 1 : 1.618) in both groups,
3. to compare the difference between facial parameters representing facial height and width in both groups.

## 2. Methods

The study comprised two groups. First group consisted of 83 female subjects between 22 and 28 years of age who were selected from the population of medical and dental students at the University of Belgrade, and the second group included 24 attractive celebrity females (popular models and actresses). Celebrities whose photos were used in this study were mostly models and actresses whose facial beauty was studied closely by the experts in the field of plastic surgery, and among them are those who were named as most beautiful and most proportional faces by the beauty and fashion magazines (such as Vogue, Cosmopolitan, and New Woman). The en face facial photographs were taken in natural head position (NHP), using camera Canon Power Shot G6, 7.1 MP, with the same distance of 1.2 m. After training and calibration, all measurements on photographs were performed by the first author (Jovana Milutinovic). In order to test the feasibility and reproducibility of the measurements, 12 photographs (10% from each group) were selected and reassessed by the same author, two months after the initial assessment. Therefore, to evaluate intra-observer agreement, Cohen's Kappa test was applied following the instructions by Landis and Koch [21].

The soft tissue points used for obtaining linear distances which were measured are shown in Table 1.

**Figure 1:**
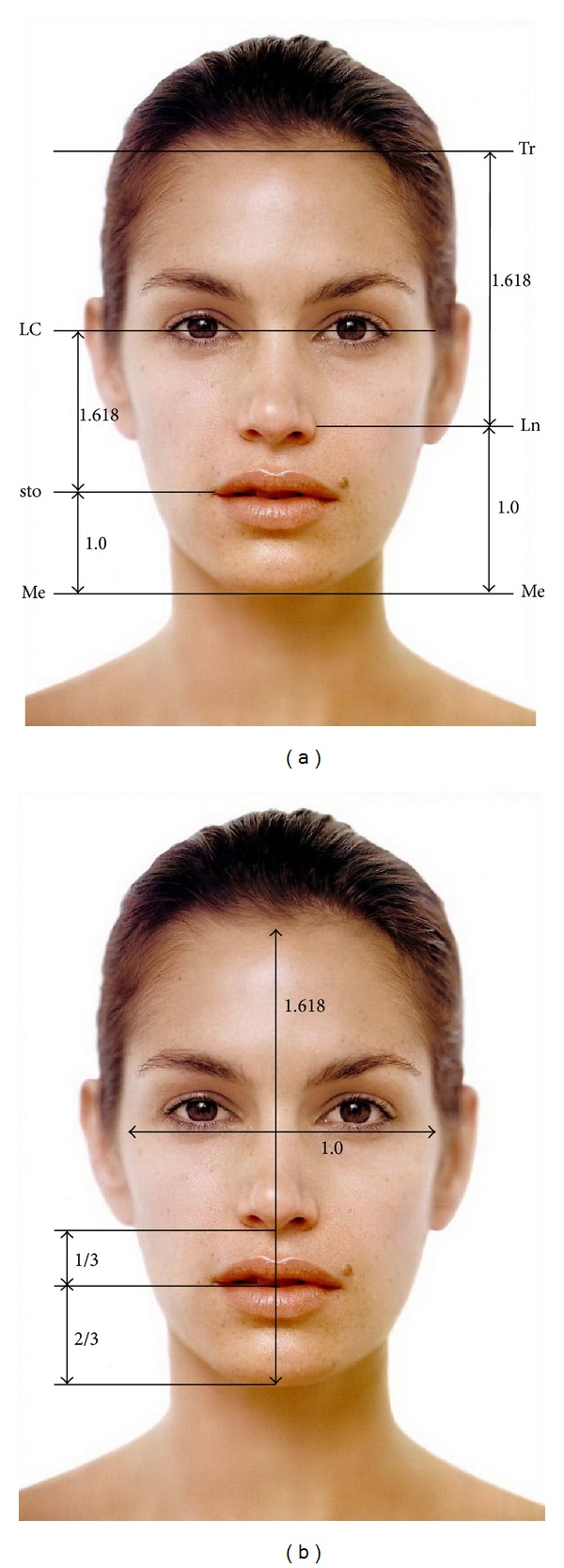
Lengths of the face and set of ideal proportions.

**Figure 2:**
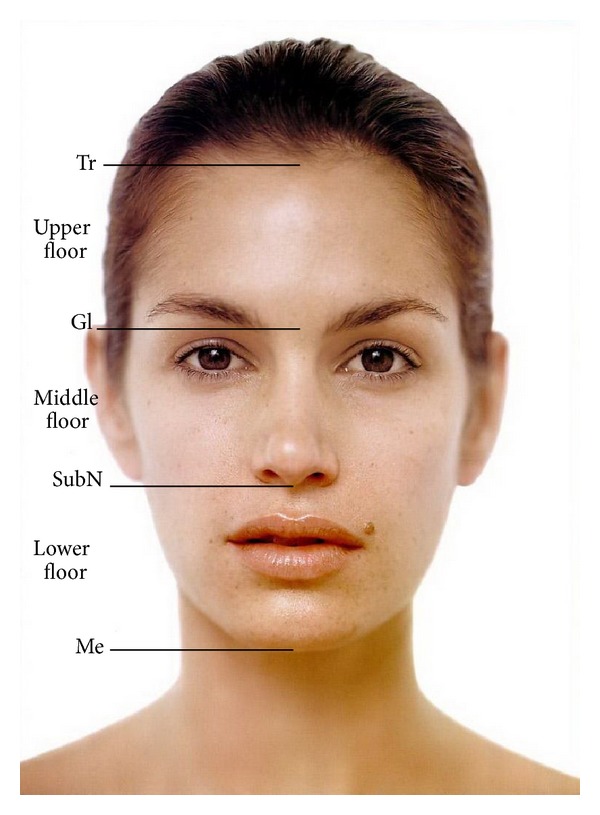
Division of the face into horizontal thirds.

**Figure 3:**
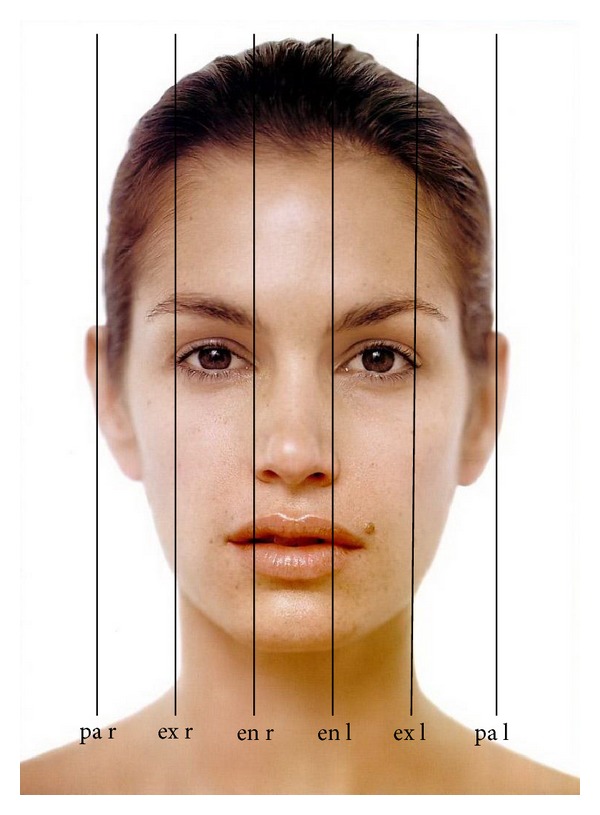
Division of the face into vertical fifths.

In the photographs, the following parameters were measured:

1. lengths of the face (Figures 1(a) and 1(b)):
  - (Tr-Me): height of the face,
  - (lchk r-lchk l): width of the face,
  - (Me-sto): the lowest point on the chin and the point where the upper and lower lip merge,
  - (sto-LC): the point where the upper and lower lip merge and corner of the eye,
  - (Me-Ln): the lowest point on the chin and the outer edge of the nostril,
  - (Ln-Tr): the outer edge of the nostril and highest point of the forehead;
2. division of the face:
  - the horizontal thirds of the face (Figure 2):
    - upper third: Tr-Gl,
    - middle third: Gl-subN,
    - lower third: subN-Me;
  - vertical fifths of the face (Figure 3)
    - pa r-ex r,
    - ex r-en r,
    - en r-en l,
    - en l-ex l,
    - ex l-pa l;
3. the ideal proportions: after marking and connecting points needed to obtain adequate lengths, measured parameters were compared with the ideal set of proportions (1 : 1.618):
  - the ratio lchk r-lchk l : Tr-Me is expected to be 1 : 1.618 (Figure 1(b)),
  - the ratio sto-Me : sto-LC is expected to be 1 : 1.618 (Figure 1(a)),
  - the ratio Me-Ln : Ln-Tr is expected to be 1 : 1.618 (Figure 1(a)),
  - the ratio subN-sto : subN-Me should be 1 : 3 [19], lower facial third index, that could also be shown in percentage (30 : 70%) [22] (Figure 1(b)).

For each and every parameter the ratio between them was used, so that the actual length of the measured parameters was of no importance.

## 3. Statistical Analysis

Statistical analyses were performed using SPSS for Windows, version 15.

The Kolmogorov-Smirnov test was applied in order to test whether the data distribution fits probability density function also known as Gaussian function or bell curve. Subsequently, if test had not rejected the assumed normal distribution, the parametric tests would have been used. For testing the differences in all parameter values between groups, independent sample *t*-test was used. For analyzing the similarity of vertical thirds and horizontal fifths of the face, in each group one-way ANOVA test was applied and in cases where ANOVA showed statistically significant difference between parameters post hoc Bonferroni test for multiple comparison was applied. To compare differences between ideal proportions and obtained proportions of the facial parameters, the authors applied paired samples *t*-test which analyzed the both values in each subject according to the concept where every particular value has its own paired “control” value. In all analyses, the significance level was set at 0.05.

## 4. Results

The Kolmogorov-Smirnov test showed normality of distribution of the obtained data in both groups. The Kappa coefficient ranged from 0.715 to 0.899 which is considered to be substantial to almost perfect agreement [21].

The mean measurement values representing length parameters of the face for both groups are shown in Table 2 as well as the differences between two groups. Almost all parameters were significantly smaller in the group of attractive females.

Tables 3(a) and 3(b) show the divisions of the face into horizontal thirds and vertical fifths for both groups. One-way ANOVA showed difference between horizontal thirds and between vertical fifths in the group of anonymous females, while in the attractive females group facial thirds and fifths were equal, with no statistical difference.

Using multiple comparison test in the group of anonymous females (Tables 3(a) and 3(b)), distance postaurale-exocanthion (pa-ex), or the most lateral fifth of the face presented by the earlobe section of the face, was found to be significantly smaller than medial three vertical fifths of the face. However, 1st and 5th vertical fifths were not significantly different one from another. Likewise, the significant difference for the middle third in comparison with the 1st and 3rd thirds of the face was found in the group of anonymous females as it was also significantly smaller. There was no significant difference between 1st and 3rd thirds.

Parameters representing division of the lower third of the face into two lengths, upper distance from the point subnasale to stomion, or the thickness of the upper lip, and lower distance from the point stomion and menton, which are supposed to be in relation 1/3 : 2/3, satisfied this criterion in the group of attractive females but not in the group of anonymous females.

Table 4 refers to the comparison of the differences between ideal proportions and obtained proportions of the facial parameters. To compare these values, the authors applied paired samples *t*-test. All analyzed parameters were found to be statistically different from the ratio 1 : 1.618 in the group of anonymous females. However, in the group of attractive females, three out of six parameters (subN-sto, sto-LC, and Ln-Tr) correspond to ideal ratio.

## 5. Discussion

The aim of this investigation was to evaluate the difference in facial proportions between attractive and anonymous Caucasian females.

Comprehension and analysis of facial parameters are necessary in different fields of medicine and dentistry, especially among specialists like plastic surgeons, maxillofacial surgeons, orthodontists, and prosthodontists [23, 24].

Keeping that in mind, there is a need for clinicians who work in a maxillofacial region to understand and become familiar with guidelines for esthetic standards and parameters of the soft tissue [22, 25, 26].

Bashour [10] found that there are four most important cues determining attractiveness: averageness, sexual dimorphism, youthfulness, and symmetry. He pointed out that a surgeon who is planning facial cosmetic, plastic, or reconstructive surgery can potentially gain both profound comprehension and better quality surgical results by appreciating these findings.

Division of the face into thirds and fifths is commonly used photogrammetric method for assessing facial symmetry. In our study, all of these measures were uniform in the group of attractive females. In 2009, Sforza et al. examined the difference between two groups of women, 24 attractive ones with 71 “normal” (healthy reference women), and obtained similar results [6]. Attractive females had several “neonatal” characteristics, such as relatively large forehead and a rounded and smaller face in general; they stated that “babyness” is the characteristic that separates them from the normal group. In our research, attractive females also had a smaller face, considering majority of parameters of the face.

Mack [27] was the first to demonstrate the practical application of ideal proportions for improving facial aesthetics. He discussed the importance of treating the dentition to the face based on the divine proportion. According to him, the lower 1/3 of the face significantly influences facial appearance. As proof, he stresses the public's preoccupation with fullness of the lips and the importance of a pleasing smile. These so-called Vitruvian thirds [22] in the lower face have to be adjusted to a 30% upper lip, 70% lower lip-chin proportion. In our study, attractive group showed harmonized lower third of the face, with lower facial index (subnasale-stomion, stomion-menton) in accordance with this beauty cannon (30% : 70%). Therefore, this ideal ratio should be suitable in planning concept for treatment in facial region [22]. These distances and divisions in the lower third of the face are one of the most important in the evaluation of facial beauty, given the fact that the lips and the chin highly determinate female beauty [12, 28].

Women lips are very impressionable feature of the face and have a strong influence on facial beauty perception. Various studies (Bisson and Grobbelaar, 2004, Ward, 1989, Torsello et al., 2010, Mommaerts and Moerenhout, 2010, and Anic-Milosevic, 2010), in which authors analyzed the lower third of the face and the lips, stated that these are one of the five important characteristics in female facial aesthetics [29–32].

Ferrario et al. (1995) reported that attractive women share several similar characteristics, such as increased upper facial third (forehead), smaller face, and more voluminous (thicker) lips than nonattractive ones. In addition, it was stressed that the length of the nose was therefore smaller in attractive group. In their research, they compared 10 attractive and 40 normal women and stated that facial characteristics of attractive females showed uniformity, while facial parameters in a normal group differed from ideal proportions [24]. In the study of Hall et al. [33] it was shown that thickness of the lips was one of the main features in the beauty perception based on a poll among orthodontists and lay public. Perseo [34] stated that, in some cases, standard camera distortions in cinema images made certain female faces appear more beautiful because they are overall “shortened.”

The studies of several authors have obtained the same results [35–37].

In the present investigation, the values for vertical length parameters, such as distance between points Me-ch and ch-LC as well as Me-Ln and Ln-Tr, which should be in relation determined by ideal proportions, differed in the group of anonymous female group. Therefore, faces of famous attractive females who represent contemporary canons of beauty are closer to the ideal proportions [5].

The question that always seems to intrigue scientists dealing with facial beauty is timelessness of the beauty principles established centuries ago. Torsello et al. (2010) found that some of the neoclassical canons can be considered still valid, while others seem to be changed over centuries. According to their research, it seems that reductions in facial medium third, in distance between eyes, and in nose dimensions have occurred as well as relative enlargement of eyes and mouth width [31].

Mommaerts and Moerenhout (2010) showed in their research that some of the ancient and neoclassical canons of beauty are still unchanged, despite the fact that some of these canons were established 2500 years ago.

These guidelines considering facial beauty can be used for improving patient's facial appearance. Clinicians must be aware that each and every person has their own beauty perception, so these results should be viewed with caution.

## 6. Conclusions

Facial beauty and its determiners are one of the most arguable topics among surgeons, dentists, and orthodontists. They can all agree about some objective guidelines concerning facial proportions, symmetry, and ratio between specific facial parameters. However, more subjective understanding of beauty is still immeasurable and lies in the eye of beholder.

This study showed significant difference between anonymous and attractive females. Attractive females showed smaller face in general and uniformity of the facial thirds and fifths, and most of the facial parameters meet the criteria of the ideal proportions.

## Figures and Tables

**Table 1:** Soft tissue points.

Point	Clarification
Trichion (Tr)	The beginning of the forehead when one lifts the eyebrow
Glabella (Gl)	The most prominent point of the forehead at the superior aspect of the eyebrows
Subnasale (subN)	Point in the midsagittal plane where the nasal septum merges into the upper lip
Menton (Me)	The most inferior point on the soft tissue chin
Stomion (sto)	Midpoint of the intralabial fissure
Postaurale (pa)	The most posterior point on the helix (outer rim of the ear)
Exocanthion (ex)	Most lateral point of the palpebral fissure at the outer canthus of the eye
Endocanthion (en)	Most medial point of the palpebral fissure at the inner canthus of the eye
Cheilion (ch)	Corner of the mouth
Lateral canthus (LC)	Lateral canthus of the eye
Lateral nose (Ln)	Lateral side of the nose
Lateral cheek (lchk)	Lateral border of the cheeks

**Table 2:** Length parameters of the face for attractive and anonymous females.

	Group	Mean (mm)	Std. dev.	Std. error mean	*t*-test
Trichion-glabella	Anonymous females	47.4759	6.90568	0.75800	0.144
	Attractive females	43.7500	11.57302	2.36233	

Glabella-subnasale	Anonymous females	44.6506	6.77366	0.74351	0.010
	Attractive females	38.1458	10.97772	2.24082	

Subnasale-menton	Anonymous females	49.6084	6.59039	0.72339	0.001
	Attractive females	42.5625	8.78402	1.79303	

Subnasale-stomion	Anonymous females	17.2169	2.47573	0.27175	0.001
	Attractive females	14.2708	3.69776	0.75480	

Menton-stomion	Anonymous females	32.4398	4.58816	0.50362	0.002
	Attractive females	28.3333	5.43472	1.10936	

Stomion-lateral canthus	Anonymous females	50.8735	7.18393	0.78854	0.071
	Attractive females	45.9375	12.26635	2.50386	

Menton-lateral nose	Anonymous females	57.3675	7.92229	0.86958	0.001
	Attractive females	48.6250	10.33067	2.10874	

Lateral nose-trichion	Anonymous females	84.3554	11.91293	1.30761	0.056
	Attractive females	75.7500	20.16292	4.11574	

Lateral nose-lateral nose	Anonymous females	28.2048	3.79830	0.41692	0.006
	Attractive females	23.9792	6.68789	1.36516	

Cheilion-cheilion	Anonymous females	38.5241	4.89145	0.53691	0.174
	Attractive females	35.8125	9.14237	1.86618	

Lateral canthus-lateral canthus	Anonymous females	72.7952	9.30547	1.02141	0.065
	Attractive females	66.1250	16.25894	3.31884	

Lateral cheek-lateral cheek	Anonymous females	97.0120	11.25209	1.23508	0.132
	Attractive females	90.2083	20.56533	4.19788	

Trichion-menton	Anonymous females	141.7229	18.82530	2.06634	0.013
	Attractive females	124.4583	30.17086	6.15860	

**Table:** (a)

Horizontal thirds						
	Mean	Std. dev.	ANOVA	Post hoc multiple comparison Bonferroni test		Sig.
Anonymous females						
1	47.4759	6.90568		1 versus 2	5.60417	0.162
2	44.6506	6.77366	0.000	1 versus 3	1.18750	0.919
3	49.6084	6.59039		2 versus 1	5.60417	0.162
				2 versus 3	4.41667	0.319

Attractive females						
1	43.75	11.57302				
2	38.1458	10.97772	0.158			
3	42.5625	8.78402				

1: trichion-glabella, 2: glabella-subnasale, and 3: subnasale-menton.

**Table:** (b)

Vertical fifths						
	Mean (mm)	Std. dev.	ANOVA	Post hoc multiple comparison Bonferroni test		Sig.
Anonymous females						
1	16.9096	2.58884		1 versus 2	7.21687*	0.000
				1 versus 3	7.07229*	0.000
				1 versus 4	7.36145*	0.000
				1 versus 5	−1.21084	0.114

2	24.1265	3.37841		2 versus 1	7.21687*	0.000
				2 versus 3	0.14458	0.998
				2 versus 4	−0.14458	0.998
				2 versus 5	6.00602*	0.000

3	23.9819	3.52296	0.000	3 versus 1	7.07229*	0.000
				3 versus 2	−0.14458	0.998
				3 versus 4	−0.28916	0.978
				3 versus 5	5.86145*	0.000

4	24.2711	3.38243		4 versus 1	7.36145*	0.000
				4 versus 2	0.14458	0.998
				4 versus 3	0.28916	0.978
				4 versus 5	6.15060*	0.000

5	18.1205	3.20403		5 versus 1	−1.21084	0.114
				5 versus 2	6.00602*	0.000
				5 versus 3	5.86145*	0.000
				5 versus 4	6.15060*	0.000

Attractive females						
1	21.7083	4.94737				
2	22.5833	5.66965				
3	21.3125	5.43302	0.947			
4	22.1208	5.63938				
5	22.0833	5.58271				

1: postaurale right-exocanthion right, 2: exocanthion right-endocanthion right, 3: endocanthion right-endocanthion left, 4: endocanthion left-exocanthion left, and 5: exocanthion left-postaurale left.

*Statistical significance.

**Table 4:** The differences between ideal proportions and obtained proportions of the facial parameters.

		Paired differences between measured and ideal values		Paired samples *t*-test
	Group	Mean (mm)	Std. dev.	Sig.
Subnasale-stomion	Attractive females	0.087	1.332	0.751
	Anonymous females	0.685	1.253	0.000

Stomion-lateral canthus	Attractive females	0.100	6.549	0.941
	Anonymous females	−1.597	4.655	0.003

Lateral nose-trichion	Attractive females	−2.929	9.031	0.126
	Anonymous females	−8.466	7.995	0.000

Lateral canthus-lateral canthus	Attractive females	3.358	3.953	0.000
	Anonymous females	−1.041	4.390	0.034

Cheilion-cheilion	Attractive females	−2.983	3.353	0.000
	Anonymous females	−7.112	3.192	0.000

Trichion-menton	Attractive females	−21.500	7.251	0.000
	Anonymous females	−15.237	6.669	0.000
